# Teflon cylindrical phantom for delivery quality assurance of SBRT

**DOI:** 10.1120/jacmp.v15i1.4536

**Published:** 2014-01-06

**Authors:** Danielle W. Lack, Ali Kakakhel, Ross Starin, Michael Snyder

**Affiliations:** ^1^ Gershenson Radiation Oncology Center Karmanos Cancer Center Detroit MI; ^2^ Department of Radiation Oncology Wayne State University School of Medicine Detroit MI USA

**Keywords:** SBRT QA, Teflon, RapidArc, TomoTherapy

## Abstract

At our institution the standard delivery quality assurance (DQA) procedure for tomotherapy plans is accomplished with a water‐equivalent phantom, EDR2 film, and ion chamber point‐dose measurements. Most plans deliver at most 5 Gy to the dose plane; however, recently a stereotactic body radiotherapy (SBRT) protocol has produced plans delivering upwards of 12 Gy to the film plane. EDR2 film saturates at a dose of ∼7Gy, requiring a modification of our DQA procedure for SBRT plans. To reduce the dose to the film plane and accommodate a possible move to SBRT using Varian RapidArc, a Teflon phantom has been constructed and tested. Our Teflon phantom is cylindrical in shape and of a similar design to the standard phantom. The phantom was MVCT scanned on the TomoTherapy system with images imported into the TomoTherapy and Varian Eclipse planning systems. Phantom images were smoothed to reduce artifacts for treatment planning purposes. Verification SBRT plans were delivered with film and point‐dose benchmarked against the standard procedure. Verification tolerance criteria were 3% dose difference for chamber measurements and a gamma pass rate >90% for film (criteria: 3 mm DTA, 3% dose difference, 10% threshold). The phantom sufficiently reduced dose to the film plane for DQA of SBRT plans. Both planning systems calculated accurate point doses in phantom, with the largest differences being 2.4% and 4.4% for TomoTherapy and Rapid Arc plans. Measured dose distributions correlated well with planning system calculations (γ<1for>95%). These results were comparable to the standard phantom. The Teflon phantom appears to be a potential option for SBRT DQA. Preliminary data show that the planning systems are capable of calculating point doses in the Teflon, and the dose to the film plane is reduced sufficiently to allow for a direct measured DQA without the need for dose rescaling.

PACS numbers: 87.56.‐V, 87.56.N‐, 87.55.‐X, 87.55.Qr.

## I. INTRODUCTION

Stereotactic body radiation therapy (SBRT) is an emerging treatment method used as an ablative therapy for tumors in the spine, chest, abdomen, and pelvis.^1^, ^2^ For many of these disease sites, local recurrence is common following conventional radiotherapy. This suggests increased doses are needed for improved local control.^(3)^ In SBRT, dose escalation is achieved in relatively few (1‐5) fractions, as biologically high doses of radiation much greater than those used in conventional fractionation schemes are used. These doses can range between as little as 6 Gy per fraction to as high as 20‐30 Gy per fraction.^2^, ^3^, ^4^ At our institution, patients with inoperable early stage non‐small cell lung cancer are treated using an SBRT schedule of 48 Gy delivered in four treatments of 12 Gy per fraction. Currently, these treatments are delivered on a helical tomotherapy (HT) system, but could in the future be delivered via Varian RapidArc (RA) therapy. The high dose per fraction used in these plans present some unique challenges for patient specific delivery QA (DQA).

The verification procedure of patient HT treatments is built into the TomoTherapy Hi‐Art II platform and has been previously discussed in detail.^5^, ^6^ In brief, it consists of EDR2 film and ion chamber measurements performed within a cylindrical water‐equivalent phantom, called the cheese phantom. Delivery accuracy is assessed by comparing relative film and absolute point‐dose measurements taken with ion chambers to those expected from the TomoTherapy treatment planning system (TPS). In SBRT plan verification, for a 12 Gy prescription dose, the dose delivered to the film plane using the current DQA procedure can reach 10 Gy, exceeding the ∼7Gy saturation point of EDR2 film.^(7)^ Currently, our solution for the overexposure of the QA film is to create and deliver two DQA plans. In the first plan, the fractional prescription dose is scaled by 50% within the TPS to 6 Gy, allowing the EDR2 film to measure the dose distribution accurately. For the second plan, the full 12 Gy prescription dose is delivered and only absolute dose to the ion chambers is measured. Use of the scaling factor for the first DQA plan changes all of the multileaf collimator (MLC) leaf opening times (LOTs) used for delivery. This may affect agreement between measured and expected dose distributions. Caution is especially advised for plans with short segment times, which after scaling may be less than the minimum LOT setting for the machine, eliminating them from delivery altogether.^(5)^ Regardless of segments being dropped, reduction of LOT times in general may cause dose discrepancies. A study by Westerly et al.^(6)^ found that plans with small LOTs were more susceptible to DQA failure, and that dose discrepancies were caused by deviations between the actual leaf latency times and those approximated by the planning system.

The HT delivery time for SBRT plans can approach 25 minutes. With our current solution, the DQA procedure for a single SBRT plan can take upwards of an hour. Aside from time considerations and failing DQA results, the changing of the MLC LOTs calls into question the applicability of the DQA using rescaled 6 Gy per fraction plans. These scaled plans are not the same as those delivered to the patient during treatment, and it is currently unknown if these modified plans are a good surrogate for the patient treatment plan. This is a concern for any IMRT delivery, but especially for SBRT treatments, for which delivery accuracy is held to a higher standard. This concern has led others to move away from the standard HT verification procedure and develop new procedures such as the ScandiDose Delta^4^ cylindrical 3D phantom (ScandiDose, Uppsala, Sweden).^(8)^ For simplicity as well as cost effectiveness, it was desirable for us maintain the same HT verification procedure. Therefore, to improve DQA efficiency and avoid dose rescaling in SBRT DQA plans, we sought to design a high‐density phantom that would reduce the dose to the EDR2 film plane, preventing film overexposure. Use of a high‐density phantom calls in to question the accuracy of which the convolution algorithms used in the Varian Eclipse and Helical TomoTherapy treatment planning systems can achieve. The ability of various dose calculation algorithms to calculate dose in high‐density media has been studied previously, with discrepancies observed. However, all of these studies have investigated the situation of a high‐density slab embedded in a solid water phantom.^9^, ^10^, ^11^, ^12^ In this work, the development and testing of a new Teflon phantom for verification of unscaled SBRT treatment plans is presented.

## II. MATERIALS AND METHODS

### A. Phantom design

The objective of the phantom design was a brute‐force reduction of dose to the phantom film plane via radiation attenuation. To adequately attenuate the overall fluence of SBRT plans, either an extremely large water‐equivalent phantom was necessary, or a phantom constructed from a higher density material. To create a phantom of manageable size (if not mass), a high‐density design using Teflon (relative electron density of 1.867 cm^−3^, mass density of 2.2 g/cm^3^) was implemented.^*^, ^†^

**Figure 1:**
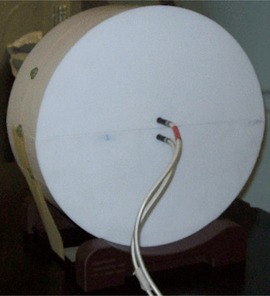
Cylindrical Teflon phantom.

The phantom is cylindrical, with a radius of 15 cm and a length of 10 cm (Fig. 1). It is cut into two semicylindrical halves, allowing for film placement along its central axis. Two holes, located 5 mm above and 10 mm below the film plane, were drilled into the phantom, enabling insertion of two Exradin A1SL ion chambers (Standard Imaging, Middleton, WI) for absolute dose measurement.

### B. Phantom commissioning

To maximize dose calculation accuracy in the DQA procedure, a proper CT‐number calibration curve must be applied,^(13)^ as discrepancies in HU values used have been shown to result in dose calculation differences of up to 3% for the HT TPS.^(14)^ Due to the high density of the phantom material, extended CT‐number calibration curves were generated for the TomoTherapy Hi‐Art II (TomoTherapy Inc., Madison, WI) and Eclipse v.8.6 (Varian Medical Systems, Palo Alto, CA) planning systems. An electron density CT phantom (Gammex RMI, Middleton, WI) with plugs of varying density including Teflon and copper was imaged on a Sensation Open CT scanner (Siemens, Malvern, PA) and TomoTherapy Hi‐Art II treatment machine to generate the KVCT and MVCT extended CT calibration curves, respectively. The average CT number values in each image set for the Teflon plugs was assigned to the density values for Teflon in the respective planning systems. Comparatively, the MVCT images of the calibration phantom were much more uniform with fewer high‐density streak artifacts than the KVCT images. The significant streak artifacts present in the KVCT images made obtaining accurate HU values for each density plug difficult. Additionally, when the Teflon phantom itself was scanned, the artifacts were even more pronounced in the KVCT image set. Therefore, the KVCT images and associated calibration curve were discarded, and only the MVCT images and curve were used for calculations in both planning systems.

The accuracy of the generated calibration curves for each TPS was evaluated by creating test plans on the Teflon phantom MVCT image set. A simple cylindrical PTV with 9 cm diameter was contoured in the center of the phantom. A dose of 20 Gy in 10 fractions was prescribed to the PTV. Calibration RA and HT plans were optimized in each respective planning system and delivery procedures were created. RA plans were delivered on a Varian Clinac iX linear accelerator (Varian Medical Systems), while HT plans were delivered on a Hi‐Art II TomoTherapy system. Film and ion chamber measurements were conducted using the Teflon phantom DQA procedure (as described in the next section) to verify the dose delivered against the dose calculated from each TPS.

It was initially found that straightforward extension of these curves using accepted density values for Teflon reported in the literature produced calculated doses inconsistent with what was measured during delivery. This inconsistency was thought to be due to, among other factors: i) heavy image artifacts preventing accurate physical representation of the phantom in the image set, ii) shifted calibration values representative of general planning system inaccuracy when calculating in such high‐density media, or iii) specific, fundamental planning system inaccuracy related to lateral scatter issues in high‐density media. Issues i) and ii) were directly addressable and could be eliminated through image processing and repeated measurement to determine those density values, realistic or not, that would allow for the respective treatment planning systems to accurately calculate dose.

To more accurately represent the phantom in each TPS and to remove unphysical image artifacts, MVCT images of the phantom were smoothed using a commercial mathematical package (MATLAB R2009b; The MathWorks, Inc., Natick, MA). The concept of applying postprocessing correction methods to CT images for the potential of improving dose calculation accuracy has been described previously.^15^, ^16^ In this work, a script was written which homogenized the varying pixel values of the Teflon phantom by setting them to an average pixel value of a representative slice. The representative slice was chosen to be one that displayed the two chamber positions and the film plane clearly. The process performed by the script on the MVCT image is summarized below:

Find an average of those pixels values that represent the Teflon phantom.
2. Determine a cutoff value such that pixels with values above this cutoff were considered to be Teflon using the representative slice.
3. Determine the average and standard deviation of all values above this cutoff value in the representative slice.
4. Replace all values within 3.25 standard deviations of that average with this average pixel value over the whole phantom.
5. Fix the obvious outlying values that were missed by the script.

**Figure 2:**
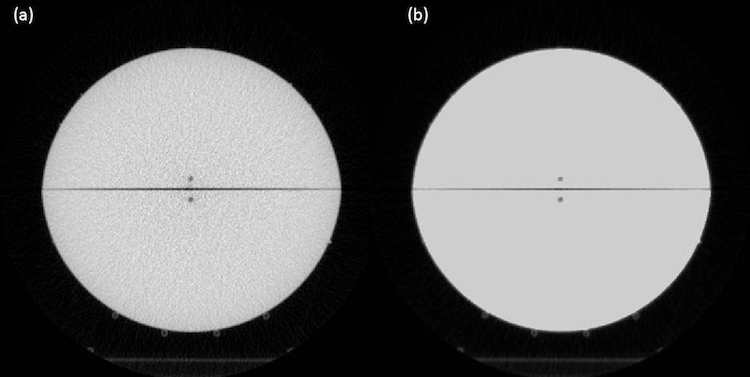
Unprocessed slice (a) of the Teflon phantom in the planning system; (b) the same image after image processing to remove artifacts.

The smoothing script determined the average MVCT HU value to be 888. The image set was therefore smoothed such that all pixel values inside the boundary of the phantom, excluding air gaps, were set to this average number. Figure 2 displays both unsmoothed and smoothed representative slices from the image sets. Repeated ion chamber measurements of the above described calibration plan were then made in the Teflon phantom to determine the delivered doses. These delivered doses were compared to the calculated dose from each planning system and the CT calibration curves were iteratively adjusted. Measured and calculated doses were found to be in good agreement when an electron density value of 1.803 cm^−3^ and a physical density value of 1.964 g/cm^3^ were correlated to the average HU of 888 in the Eclipse and TomoTherapy planning systems, respectively.

### C. SBRT plan DQA

Six SBRT patients with peripherally located NSCLC previously treated on TomoTherapy were evaluated in our DQA test procedure. The range of PTV volumes treated for these six patients was 7.63‐83.43 cc. For each patient, RA SBRT plans were created retrospectively in the Eclipse TPS. RapidArc plans consisted of two full arcs, and used the same prescription of 48 Gy delivered in 4 fractions to 95% of the PTV volume. All plans were optimized similarly to existing TomoTherapy plans used for treatment using Eclipse's progressive resolution optimizer (PRO).

DQA plans were generated for both RA and HT patient treatments by applying a forward dose calculation of each plan onto both the smoothed and unsmoothed MVCT image sets of the Teflon phantom, allowing the effect of imaging smoothing on DQA results to be determined. This was conducted in each respective TPS, using either the DQA protocol in TomoTherapy or the verification plan protocol in Eclipse. These protocols differ in that for TomoTherapy, a newly calculated DQA plan is delivered, while in Eclipse, the verification plan calculated is only used for comparison (while the patient plan is used for delivery). DQA measurements for both sets of SBRT plans were conducted using Kodak EDR2 film (Eastman Kodak Company, Rochester, NY) and two A1SL ion chambers inserted into their respective holes in the phantom. When determining the expected ion chamber doses in both the Eclipse and TomoTherapy treatment planning systems, several dose points were chosen over each chamber volume. These dose points were averaged and used for comparison with the chamber measurements obtained. Ion chamber measurements were determined by acquiring a charge measurement and applying appropriate ion chamber correction factors including the Accredited Dosimetry Calibration Laboratory (ADCL) absorbed dose‐to‐water calibration factor for $\text{Co}-60\;({N_{D,W}}^{\text{Co}-60})$ values for each chamber, the ADCL electrometer correction faction (Pelec), the temperature‐pressure correction factor ($P_{\text{TP}}$), and a beam quality conversion factor value ($k_{Q}$) of 0.998, as referenced in the American Association of Physicists in Medicine (AAPM) TG‐148 report for a percent depth‐dose at 10 cm depth with electron contamination removed $\left(\%\text{dd}({10)}_{x}\right)$ of 63.^(17)^ Film dose distributions were determined by generating an EDR2 calibration curve correlating pixel value after scanning to measured dose with a calibrated ion chamber. For consistency, film analysis for both types of plans was conducted using RIT v.5.2 film analysis software (Radiological Imaging Technology, Inc., Colorado Springs, Co). Analysis was based on the qualitative agreement of measured and planned dose distributions in the film plane and calculated gamma (γ) indices (gamma criteria: 10% threshold, 3 mm DTA, and 3% dose difference).

Mirroring the concerns about the effects of rescaling treatment plan doses, it is possible to intentionally create tomotherapy plans that will fail standard DQA by sufficiently rescaling the dose.^(6)^ Scaling the dose downward to values much lower than the original plans affects the mean leaf opening time of the MLC during delivery. When the mean leaf opening time is sufficiently small, the planning system is unable to accurately model the physical beam and dose discrepancies occur. To test the ability of the Teflon phantom to detect such errors, failing HT DQA plans were also created and delivered to the Teflon phantom. Failing plans were created by recalculating the dose for each SBRT patient plan on the Teflon phantom within the TomoTherapy DQA protocol, and then scaling the prescription dose for these plans before creating the delivery procedures. Scaling for each patient was based on the estimated mean leaf opening time, $t_{\text{mean}}$ for the original unscaled plan:

(1) $$t_{mean}=\frac{1}{MF}(\frac{T}{51})$$

where *MF* is the plan modulation factor, and *T* is the plan gantry period, and *51* represents the number of projections per rotation.^(6)^ Plans were scaled to achieve a mean leaf opening time of less than 100 ms. This was based on the Westerly et al. study,^(6)^ which showed that plans with many small LOT segments (mean leaf opening time<100ms) were more susceptible to the effects of leaf timing inaccuracies, and when verified, failed point dose measurements with dose discrepancies over 3%. For most of the HT SBRT plans evaluated in this study, scaling was on the order of ∼20% of the original prescription dose per fraction (12 Gy) or 2.4 Gy.

### D. Benchmarking of DQA test procedure

Before clinically implementing the Teflon phantom for SBRT DQA, it was of interest to benchmark this phantom against an established DQA procedure. The TomoTherapy cheese phantom with EDR2 film and A1SL ion chambers was used in the benchmarking process. A KVCT image set of the cheese phantom was acquired on a Somatom Sensation Open CT scanner and imported as a phantom into the Eclipse and TomoTherapy planning systems. Images of this phantom had minimal or no streak artifacts; therefore, MVCT images were not needed. Both the HT original and failing plans, as well as RA plans, for each patient were calculated on the cheese phantom via the same DQA and verification protocols within the two planning systems. As mentioned earlier, the EDR2 film saturates at a dose of ∼7Gy. Therefore, in order to DQA the original SBRT RapidArc and TomoTherapy plans with the cheese phantom, two DQA plans, one scaled and the other unscaled, were needed to prevent film overexposure. Only chamber measurements were performed for full dose plans, while both film and chamber measurements were performed for scaled plans. All planar dose distribution analysis was performed using the RIT v. 5.2 software platform.

### E. complexity analysis

In a typical DQA, the dose distribution as calculated in the cheese phantom is considered to be a reasonable surrogate for the actual patient dose distribution due to the roughly tissue‐equivalent nature of the phantom material (patient heterogeneities not withstanding). The Teflon phantom is, however, not tissue equivalent, and as such the dose distribution within the phantom may not be as useful a surrogate as the cheese phantom distribution. It is possible that the increased density of the Teflon phantom could smooth the dose distributions, reducing their complexity and making the Teflon‐DQA test less susceptible in general to deviations between calculated and measured highly conformal distributions. If, however, the complexity of the distribution in the Teflon phantom is equivalent to that of the distribution in the cheese phantom, it should indicate equivalence in the ability of both phantoms to act as a surrogate for the patient distribution.

To estimate the complexity of the dose distributions for each patient and phantom, we calculated and compared the entropy^18^, ^19^ of each distribution. Each image was binned into 256 grey levels uniformly distributed between the image's minimum and maximum dose. The grey level of 0, represented by black, corresponds to the minimum dose and the grey level 255, represented by white, corresponds to the maximum dose. A histogram, $\left\{p_{i}\right\}$, of the number of pixels of each grey level, *i*, was generated. Each distribution contains a set of grey levels at the low end that represents background or zero dose. This set of grey levels appears as a sharp peak at the low end of the histogram. The cutoff between the background and doses of interest was taken to be the grey level where this peak no longer continued to decline. Only grey levels at or above this cutoff were used for entropy calculations. The entropies, H, were calculated using the formula below with no bias correction:

(2) $$H=-\sum_{i}p_{i}{\text{log}}_{2}p_{i}$$

## III. RESULTS

The effect of smoothing the Teflon images on our SBRT DQA procedure is presented in Tables 1 and 2.

Without image smoothing applied to the Teflon phantom, there is less agreement between DQA measurements and doses predicted by each TPS. Differences were largest for the film analysis results, where for RA and HT SBRT plans, the average gamma pass rate improved by 4.8% and 2.0%, respectively, when image smoothing was applied. It is of note that, in addition to improving the agreement of both the absolute dose measurements and the dose plane comparisons, image smoothing also reduces the variation between the plans, and thus improves the stability and reliability of the DQA results overall.

**Figure 3:**
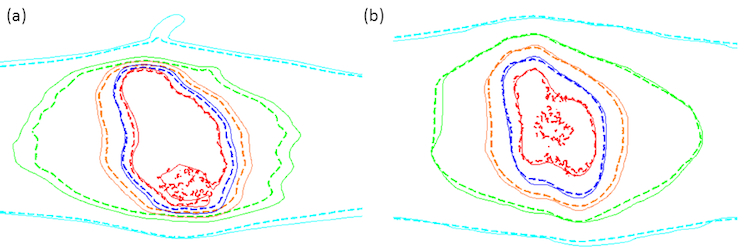
Isodose curves for a representative for a (a) RA and (b) HT DQA delivery. Solid contours represent the calculated isodose curves from each respective TPS, while the dashed contours represent the dose distribution measured in the Teflon phantom. Isodose levels shown are relative and as follows: 90% (red), 70% (blue), 50% (orange), 30% (green), and 10% (light blue).

Using the smoothed Teflon image set, DQA results for the Teflon phantom were found to be comparable or better to those obtained with the cheese phantom. Table 3 shows the absolute point dose measurements obtained for RA and HT plans on each phantom. For the Teflon phantom, the average differences between ion chamber dose measurements and calculated point doses were 0.5%±1.4% and 0.3%±1.2% for RA and HT, respectively. For the cheese phantom, average differences were 2.0%±1.8% for RA and 0.4%±1.7% for HT. For the lung‐SBRT fractionation scheme delivered at our center using 4 fractions of 12 Gy, the Teflon phantom adequately reduced the dose to the EDR2 film to prevent overexposure, as compared to the clinically employed cheese phantom procedure. The maximum measured dose at the film plane for all SBRT plans tested was 7.3 Gy. The dose distributions measured in the Teflon phantom qualitatively agreed with those calculated in both planning systems. A representative measured dose distribution in the Teflon phantom for the TomoTherapy plans and the RapidArc plans are presented in Fig. 3. Quantitatively, the measured film dose required rescaling by as much as 9% from the original film calibration, most likely as a result of differences in processing conditions and variation between individual film pieces and batches. However, after rescaling all plans had an average y‐pass rate of 98.6%±1.4% and 96.8%±2.5% for RapidArc and TomoTherapy, respectively, with no plans below the 90% threshold. The average y‐pass rates for the cheese phantom were similar (99.4%±0.8% for RA, 97.8%±2.9% for HT). A summary of the film analysis results for both the cheese and Teflon phantoms is presented in Table 4.

**Table 1:** Measured (Mx) and calculated (TPS) point doses for unsmoothed and smoothed Teflon image sets.^a^

		*Unsmoothed Image Set*		*Smoothed Image set*	
*Patient No.*	*Ion chamber Position*	*RA*	*HT*	*RA*	*HT*
		*Mx/TPS*	*Mx/TPS*	*Mx/TPS*	*Mx/TPS*
1	Upper	0.991	0.997	0.994	0.999
	Lower	0.993	—	1.002	—
2	Upper	0.995	1.018	0.999	0.986
	Lower	0.992	—	1.012	—
3	Upper	0.948	1.022	0.956	1.005
	Lower	0.992	1.029	1.000	1.024
4	Upper	0.990	0.994	1.001	1.002
	Lower	0.988	1.005	1.004	0.989
5	Upper	0.987	1.029	0.993	0.994
	Lower	0.990	1.001	1.002	0.987
6	Upper	0.977	1.009	0.983	0.992
	Lower	0.983	—	0.997	—
Average		0.985	1.011	0.995	0.997
SD		0.013	0.013	0.014	0.012

a ^a^ Dashed lines represent a chamber measurement in a high‐gradient area.

**Table 2:** Gamma pass rates for the smoothed and unsmoothed Teflon phantom.^a^

	*Unsmoothed Image Set*		*Smoothed Image set*	
*Patient No.*	RAγPass Rate(%)	HTγPass Rate(%)	RAγPass Rate(%)	HTγPass Rate(%)
1	75.6	99.0	99.8	94.9
2	99.9	86.2	99.9	92.9
3	96.6	97.2	96.2	98.2
4	97.3	97.3	98.6	97.3
5	95.0	92.2	98.4	97.8
6	98.5	96.5	98.4	99.8
Average	93.8	94.8	98.6	96.8
SD	9.1	4.7	1.4	2.5

a ^a^ Note that film rescaling to account for dosimetric variations between film pieces and processor conditions was applied in determining these results.

**Table 3:** Summary point doses for the Teflon and cheese phantoms for RA and HT DQA plans. Results are given as a ratio of the measured (Mx) point dose/expected point dose from each TPS. Teflon results are for plans calculated on the smoothed phantom image set.^a^

		*Teflon Phantom*		*Cheese Phantom*	
*Patient No.*	*Ion chamber Position*	*RA*	*HT*	*RA*	*HT*
		*Mx/TPS*	*Mx/TPS*	*Mx/TPS*	*Mx/TPS*
1	Upper	0.994	0.999	1.013	1.045
	Lower	1.002	—	1.039	0.976
2	Upper	0.999	0.986	1.016	0.992
	Lower	1.012	NA	1.060	1.008
3	Upper	0.956	1.005	1.019	1.006
	Lower	1.000	1.024	1.031	1.009
4	Upper	1.001	1.002	1.008	0.990
	Lower	1.004	0.989	1.015	1.000
5	Upper	0.993	0.994	1.000	1.013
	Lower	1.002	0.987	1.015	0.998
6	Upper	0.983	0.992	0.995	0.991
	Lower	0.997	—	1.028	1.016
Average		0.995	0.997	1.020	1.004
SD		0.014	0.012	0.018	0.017

a ^a^ Dashed lines represent a chamber measurement in a high‐gradient area.

**Table 4:** Film analysis results for the Teflon and cheese phantoms for RA and HT DQA plans. Teflon results are for plans calculated on the smoothed phantom image set. Cheese results are for DQA plans with 50% dose scaling applied.^a^

	*Teflon Phantom*		*Cheese Phantom*	
*Patient No.*	RAΥPass Rate(%)	HTΥPass Rate(%)	RAΥPass Rate(%)	HTΥPass Rate(%)
1	99.8	94.9	99.8	90.9
2	99.9	92.9	99.1	99.1
3	96.2	98.2	98.0	98.2
4	98.6	97.3	99.4	99.9
5	98.4	97.8	100.0	100.0
6	98.4	99.8	100.0	98.5
Average	98.6	96.8	99.4	97.8
SD	1.4	2.5	0.8	3.4

a ^a^ Note that film rescaling to account for dosimetric variations between film pieces and processor conditions was applied in determining these results.

For the plans that were designed to fail during TomoTherapy DQA, the average difference in the measured and calculated point doses was 6.5%±1.6% for the Teflon phantom and 7.5%±1.1% for the cheese phantom. Similarly, film results for these scaled plans were also significantly worse as compared to the unscaled TomoTherapy plans for each patient, with an average y‐pass rate of 72.8%±8.6% and 75.9%±8.5% for the Teflon and cheese phantoms, respectively. This test was only performed for TomoTherapy plans because we had no reliable way of creating plans that would fail during RapidArc delivery. However, this does not mean that RapidArc treatments are more resistant to failure from any kind of dose scaling, but that the relationship between dose scaling and failing DQA results is currently unknown.

**Figure 4:**
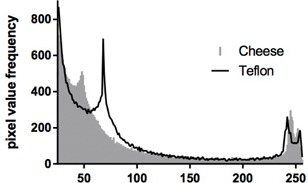
Sample entropy histogram for one patient showing the number of pixels in each grey scale level for the Teflon and cheese DQA plans.

The complexity analysis of the RA dose distributions for both the Teflon and cheese phantoms showed similar entropies for both of the phantoms (Table 5). The similar entropies of the distributions indicate similar complexities in the calculated doses, suggesting that measurements of the distributions would be equally representative of the complexity of the original patient plan. Overall the largest difference between the entropies of the two phantoms was 1.1%. Figure 4 shows a sample histogram of grey levels.

**Table 5:** Entropy values for RA DQA plans on the cheese and Teflon phantoms, respectively

	*Entropy (H)*		
*Patient No.*	*Cheese Phantom*	*Teflon Phantom*	*Teflon/Cheese Entropy*
1	4.810	4.850	1.008
2	4.708	4.720	1.003
3	4.750	4.770	1.004
4	5.133	5.175	1.008
5	5.090	5.137	1.009
6	4.821	4.876	1.011
Average	4.885	4.921	1.007
SD	0.180	0.190	0.003

## IV. DISCUSSION

It is clear from the preliminary data that, after film rescaling, the dose distributions measured in the Teflon phantom agree with those predicted by both treatment planning systems. In addition to the accurate calculation of the dose distributions themselves, the point‐dose measurements showed excellent agreement with those calculated in each planning system, provided the image sets were properly smoothed and the calibration curves carefully constructed.

Benchmarking using the accepted standard cheese phantom for DQA showed that the pass rates for the plans on the Teflon phantom were consistent with those seen with the cheese phantom. Perhaps more importantly, the Teflon phantom DQA results showed failures consistent with those seen on the cheese phantom, displaying the ability of the phantom to detect clinically unacceptable plans.

Despite the apparent initial success of implementing a high‐density phantom for DQA, questions remain with regard to the general applicability of such a phantom. The SBRT plans chosen for this study represent a reasonable cross section of plan types that would normally be seen in the clinic. However, due to the limitations of the lung‐SBRT protocol, these plans typically are designed to deliver high doses to small, roughly spherical lesions. These plans, therefore, often have limited amounts of modulation overall. Other treatment sites requiring plans with greater amounts of beam modulation will stress the ability of the planning system to calculate more complex dose distributions in ways that our initial test have not. Given the uncertainties in the results of the dose calculation algorithms in very high‐density media, it is unclear if the calibration procedures would be sufficient to produce usable DQA results for every type of treatment scenario.

In addition to limiting the results of this study to a specific lesion type, limits are also implicitly placed on the type of fractionation scheme for which the phantom presented here would be useful. Other SBRT fractionation schemes delivering upwards of 36 Gy per fraction obviously exceed the dose reductions to the film plane that could be achieved by simply attenuating the beam with denser materials. For fractionation schemes much larger than 12 Gy, it would be necessary to use other methodologies of DQA.

## V. CONCLUSIONS

The Teflon phantom appears to be a potential option for 4 fraction lung SBRT DQA. Preliminary data show that the planning systems are capable of calculating point doses in the Teflon, and the dose to the film plane is reduced sufficiently to allow for a direct measured DQA without the need for dose rescaling. While other, more costly phantoms exist for high dose per fraction DQA, the simplicity of a well‐calibrated Teflon setup, the time‐tested ion chamber point dose measurements, and the high resolution of EDR2 film, provide a potential low‐cost, accurate method of performing quality assurance for certain lung SBRT protocols.
